# Supplementary material for: A Retrospective Study on the Role of Metformin in Colorectal Cancer Liver Metastases
Source: Biomedicines. 2023 Feb 28;11(3):731. doi: 10.3390/biomedicines11030731 (PMC10045020; doi:10.3390/biomedicines11030731)
Supplement: Supplementary file 1 [file biomedicines-11-00731-s001.zip › Supplementary figures.pdf]

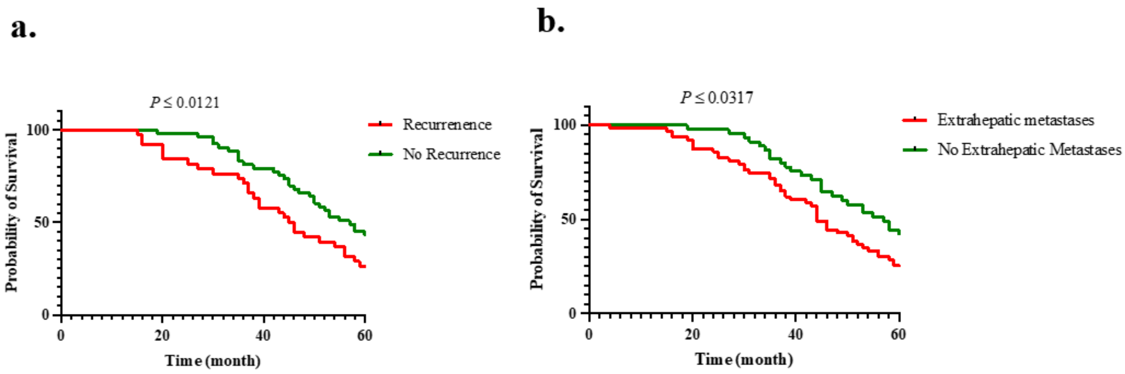

Supplementary Figure S1. The impact of recurrence and extrahepatic incidence on survival rate in CRCLM. Overall survival of CRCLM patients in the presence of recurrence (**a**) or extrahepatic incidence (**b**).

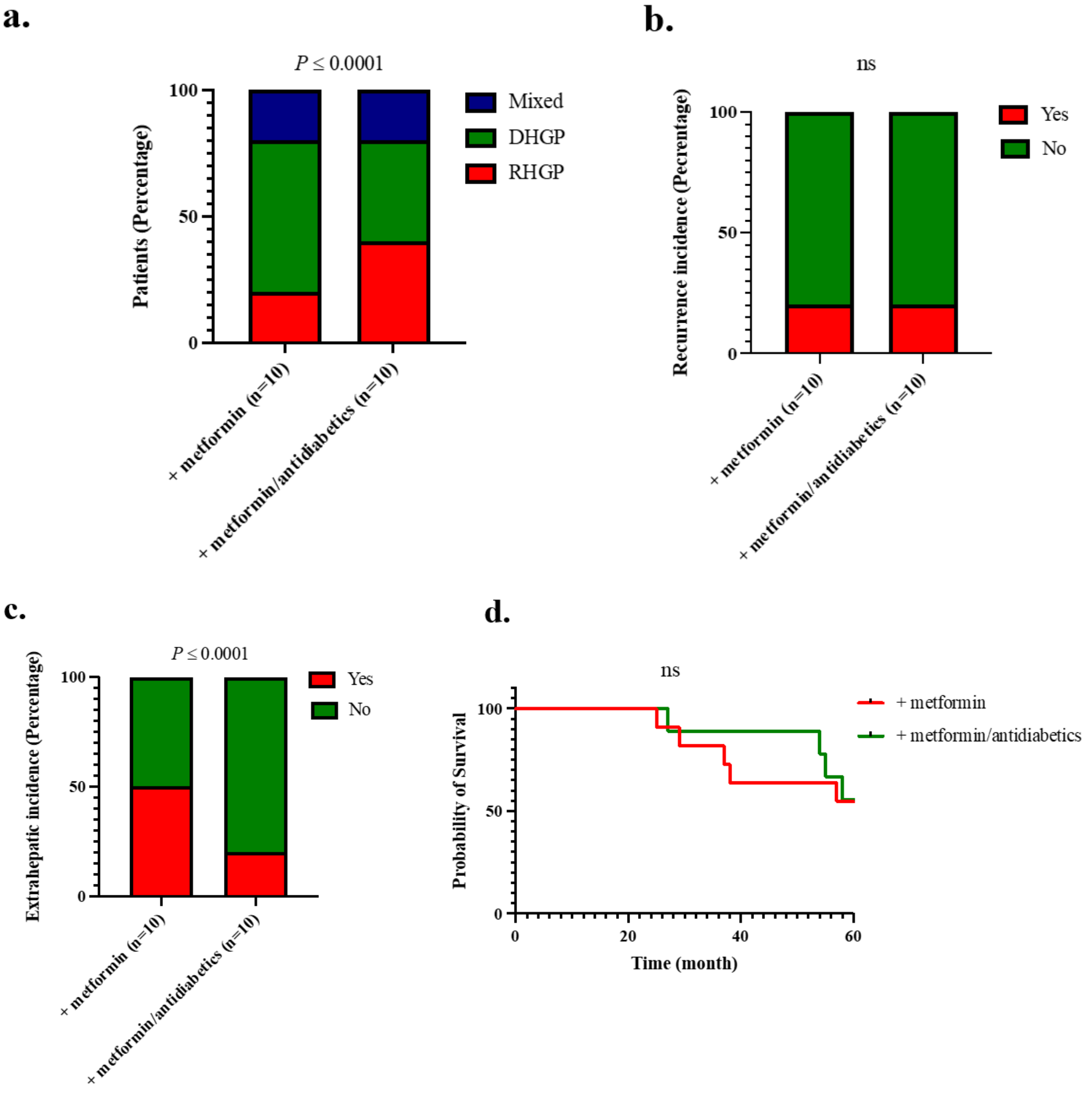

Supplementary Figure S2. The role of various antidiabetics in CRCLM. Shows the impact of metformin either individually or combined with other antidiabetics on (**a.**) tumour HGPs, (**b.**) recurrence and (**c.**) extrahepatic metastases ratios. (**d.**) Overall survival of CRCLM patients who used metformin individually or combined with other antidiabetics.
